# Development of a Sensitive UPLC-MS/MS Method for the Simultaneous Quantification of Mycotoxins in Wheat Products and Human Urine

**DOI:** 10.3390/toxins18050219

**Published:** 2026-05-06

**Authors:** Bin Gao, Jialin Sun, Zechao Xu, Xiaohui Li, Jianxin Ma, Xiaomin Han, Shuo Wang

**Affiliations:** 1 School of Public Health, Baotou Medical College, Baotou 014040, China; 2 Chaoyang District Center for Disease Control and Prevention (Chaoyang District Health Supervision Institute), Beijing 100021, China; 3 NHC Key Laboratory of Food Safety Risk Assessment, China National Center for Food Safety Risk Assessment, Beijing 100021, China; 4 School of Public Health, Southern Medical University, Guangzhou 510515, China

**Keywords:** mycotoxins, wheat products, human urine, UPLC-MS/MS

## Abstract

Mycotoxin contamination in wheat products has consistently been a key issue of concern in food safety, and urinary biomonitoring provides an effective approach for assessing internal human exposure. In this study, a sensitive ultra-performance liquid chromatography–tandem mass spectrometry method was developed and validated for the simultaneous determination of 28 mycotoxins in wheat products and human urine. For the two matrices, the extraction solvent, acid concentration, solid-phase extraction cartridge type, and enzymatic hydrolysis parameters were optimized. Under the optimized conditions, all target compounds showed excellent linear relationships within the tested concentration ranges (R^2^ > 0.99). In wheat products, the spiked recoveries ranged from 70.2% to 120%, the repeatabilities ranged from 1.6% to 9.1%, and the limits of detection and limits of quantification were 0.001~8.3 μg/kg and 0.002~25.0 μg/kg, respectively. In urine, the spiked recoveries ranged from 79.3% to 120%, the repeatabilities ranged from 0.7% to 9.4%, and the limits of detection and limits of quantification were 0.0001~1.0 μg/L and 0.0002~3.0 μg/L, respectively. Analysis of real samples showed that at least seven mycotoxins were detected in wheat product samples, and at least five were detected in urine samples. In wheat products, the detection rates of deoxynivalenol, enniatin B, enniatin A_1_, enniatin B_1_, tenuazonic acid, and tentoxin were all 100%, whereas in urine, the detection rate of fumonisin B_1_ reached 100%, and tenuazonic acid showed the highest mean concentration in both matrices. In conclusion, the developed ultra-performance liquid chromatography–tandem mass spectrometry method is suitable for the simultaneous quantification of 28 mycotoxins in wheat products and human urine, and its preliminary application demonstrates good practical applicability.

## 1. Introduction

Mycotoxins are secondary metabolites mainly generated by *Aspergillus*, *Penicillium*, *Fusarium*, *Alternaria*, and *Claviceps* molds. They are generally stable compounds and are mostly found in agricultural products and their derived food and feed products [1,2,3,4]. The Food and Agriculture Organization of the United Nations (FAO) has estimated that at least 25% of agricultural crops worldwide are affected by mycotoxin contamination each year [5]. Apart from major mycotoxin classes, so-called emerging mycotoxins such as alternariol (AOH), alternariol monomethyl ether (AME), tentoxin (TEN), tenuazonic acid (TeA), and altenuene (ALT) produced by *Alternaria* species, as well as beauvericin (BEA) and enniatins (ENNs) produced by *Fusarium* species, have received increasing attention in recent years [6,7].

Both regulated and emerging mycotoxins are widely detected in cereal crops and often occur as co-contaminants, posing a serious threat to food safety [8,9,10]. Wheat is one of the most important foods and provides a staple food source for approximately 35% of the global population [11]. China is one of the major wheat-producing countries worldwide, accounting for a substantial proportion of global wheat production, and wheat-based products, which are important dietary staples, particularly in northern China [12,13]. Recent studies have shown that deoxynivalenol (DON), zearalenone (ZEN), BEA, ENNs, and *Alternaria* toxins are frequently detected in wheat [7,14,15,16]. In 2021, 321 wheat samples collected in China were found to contain at least one mycotoxin. The detection rates of mycotoxins ranged from 7.1% to 100%, and DON and TeA showed the highest detection rates and concentration levels. The highest concentration was 921.8 μg/kg [7]. Another study by Hassine et al. on durum wheat from Tunisia showed that multiple mycotoxins, including DON, ZEN, ochratoxin A (OTA), enniatin A_1_ (ENNA_1_), enniatin B (ENNB), and enniatin B_1_ (ENNB_1_), were detected in durum wheat from Tunisia collected during 2021–2022; the detection rates ranged from 5.98% to 40.71%, with concentrations ranging from 0.01 to 384.2 μg/kg. [17].

Most mycotoxins are hepatotoxic, nephrotoxic, teratogenic, immunosuppressive and estrogenic, posing diverse health hazards to humans and animals [1,2,3,4]. Several food-contaminating fungal species have been reported to interfere with estrogenic pathways [18,19]. Among the *Alternaria* mycotoxins known to date, AOH and AME have been reported to exert not only genotoxic effects, but also to potentially act as endocrine-disrupting chemicals (EDCs) [20,21,22]. Therefore, it is critical to assess human exposure to these food contaminants.

Mycotoxin contamination in food does not fully reflect the actual internal exposure of individuals; urinary biomonitoring has become one of the major approaches for assessing mycotoxin exposure in human populations [23,24,25]. Urine is the most common biomatrix used to assess exposure to mycotoxins due to its accessibility and easy sampling. In the human body, absorbed mycotoxins are primarily excreted into the urine. As a result, both free toxins and their metabolites or conjugated forms can be detected in urine [23,26]. Some mycotoxins can be monitored directly as parent compounds in urine, such as fumonisin B_1_ (FB_1_), fumonisin B_2_ (FB_2_), fumonisin B_3_ (FB_3_), OTA, ENNA, ENNA_1_, ENNB, BEA, AOH, and TEN, whereas others are more appropriately assessed through their metabolites or conjugated forms [23]. In the case of aflatoxins, aflatoxin B_1_ (AFB_1_) can be biotransformed in the liver to aflatoxin M_1_ (AFM_1_) and subsequently excreted in urine. Therefore, AFM_1_ is considered more informative than unmetabolized AFB_1_ in urinary biomonitoring [23,27]. For DON, it is present in human urine mainly as glucuronide conjugates, and thus, urinary DON is commonly determined after enzymatic hydrolysis to measure the free form [26]. Among these, AFM_1_, DON, and deoxynivalenol-15-glucuronide (DON-15-GlcA) have been used as urinary biomarkers of exposure [23,26,28]. ENNB_1_ can undergo phase I biotransformation in humans, including hydroxylation, carbonylation, carboxylation, and N-demethylation. Both the parent compound and its metabolites may, therefore, serve as potential urinary biomarkers for exposure [29,30]. One study showed that the detection rate of AFM_1_ in urine samples from lactating women in Bangladesh was 88.9%, with a mean concentration of 109.9 ± 52.8 pg/mL and a range of 40.0 ~ 223.8 pg/mL [27].

Although various LC–MS/MS-based methods for the determination of multiple mycotoxins have been widely reported, the number of toxins covered remains limited, typically including only several or a dozen conventional mycotoxins. Moreover, the methods established to simultaneously determine both conventional and emerging mycotoxins are still relatively scarce [31,32,33,34,35]. In addition, food samples and biological samples always use different pretreatments, including different extraction solvents and different solid-phase extraction (SPE) methods. In this study, we aimed to establish a solid-phase extraction method for two matrices in order to improve detection efficiency. Meanwhile, considering that conventional mycotoxins have remained at relatively high contamination levels in recent years and that emerging mycotoxins have attracted increasing attention, it is necessary to establish a sensitive ultra-performance liquid chromatography–tandem mass spectrometry (UPLC–MS/MS) method for the simultaneous determination of 28 mycotoxins in wheat products and human urine, including AFB_1_, aflatoxin B_2_ (AFB_2_), aflatoxin G_1_ (AFG_1_), aflatoxin G_2_ (AFG_2_), AFM_1_, OTA, ochratoxin B (OTB), ochratoxin C (OTC), FB_1_, FB_2_, FB_3_, T-2 toxin (T-2), HT-2 toxin (HT-2), DON, 3-acetyldeoxynivalenol (3-Ac-DON), 15-acetyldeoxynivalenol (15-Ac-DON), sterigmatocystin (ST), cyclopiazonic acid (CPA), ENNA, ENNA_1_, ENNB, ENNB_1_, BEA, ALT, AOH, AME, TEN, and TeA. In this study, different extraction solvents were used for wheat products and human urine, while the same solid-phase extraction procedure was applied for cleanup of both matrices. Finally, the established method was applied to the analysis of real samples, providing technical support for the assessment of mycotoxin contamination in wheat products and human biomonitoring.

## 2. Results and Discussion

### 2.1. Optimization of Sample Extraction Solvent

Due to the diversity of 28 mycotoxins, with log*p* values approximately spanning about −1.4 to 5.0 and pKa values spanning 2.6 to 18.8, the extraction solvent is one of the most critical factors affecting mycotoxin extraction efficiency during sample pretreatment. To achieve the maximum extraction of multiple mycotoxins from wheat products and urine, this study compared different extraction solvents for these two matrices.

**Figure 1:**
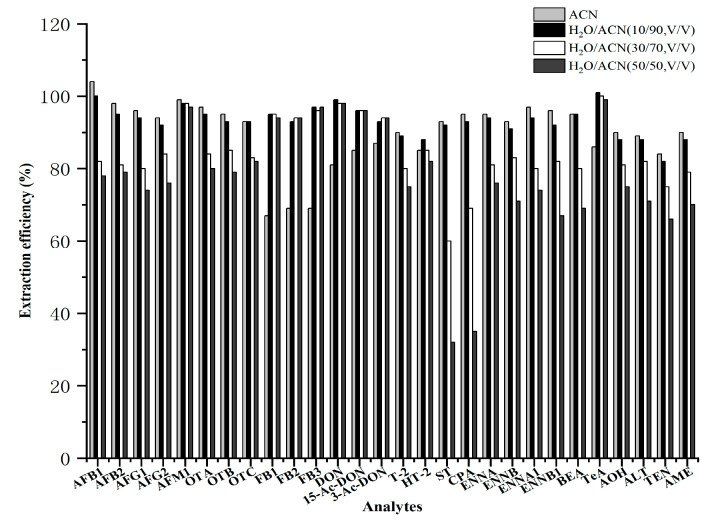
Extraction efficiencies of different extraction solvents for 28 mycotoxins in wheat products.

For the wheat product matrix, this study evaluated the efficiency of four extraction solvents, including acetonitrile (ACN), H_2_O/ACN (10/90, *v*/*v*), H_2_O/ACN (30/70, *v*/*v*), and H_2_O/ACN (50/50, *v*/*v*). The results presented in Figure 1 indicate that when H_2_O/ACN (10/90, *v*/*v*) was used as the extraction solvent, the extraction efficiencies of all mycotoxins exceeded 85%. However, when the proportion of water increased to 30% and 50%, the extraction efficiencies of aflatoxins (AFs) (except AFM_1_), ST, CPA, ENNs, and BEA were lower than those obtained with H_2_O/ACN (10/90, *v*/*v*). When using ACN as the extraction solvent, the extraction efficiencies of fumonisins (FBs) were all below 70%, which was probably related to the structure of FBs. FBs contain multiple hydroxyl and carboxyl groups in their molecules, which readily form hydrogen bonds with water and therefore show relatively high hydrophilicity; thus, a certain proportion of water in the extraction solvent was necessary to improve the extraction efficiency [36]. Therefore, H_2_O/ACN (10/90, *v*/*v*) was selected as the extraction solvent.

**Figure 2:**
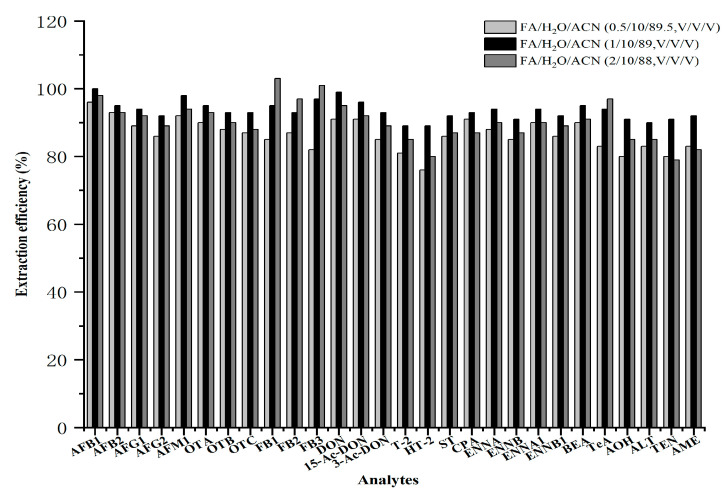
Extraction efficiencies of different formic acid concentrations for 28 mycotoxins in wheat products.

Because FBs and TeA are sensitive to acidic conditions, they are more likely to transfer from the aqueous phase to the organic phase under acidic conditions [37,38,39]. Therefore, this study compared the effects of adding three different formic acid concentrations (0.5%, 1.0%, and 2.0%) to H_2_O/ACN (10/90, *v*/*v*) on extraction efficiency. As shown in Figure 2, the addition of formic acid obviously improved the extraction efficiencies of FBs and TeA. When the formic acid concentration increased from 0.5% to 1.0%, the extraction efficiencies of all toxins were higher than 89.0%. However, when the formic acid concentration increased to 2.0%, the extraction recovery of all other compounds, except FBs and TeA, decreased. The recovery of TEN decreased from 91.0% to 79.0%. Overall, H_2_O/ACN (10/90, *v*/*v*) containing 1.0% formic acid was finally selected as the extraction solvent.

**Figure 3:**
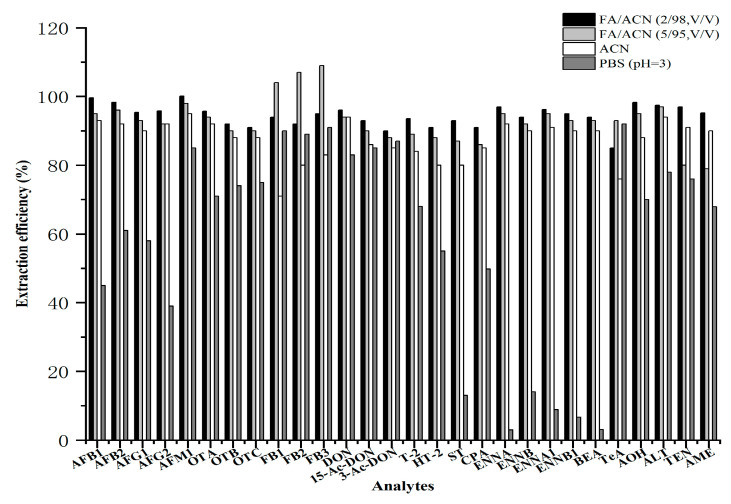
Extraction efficiencies of different extraction solvents for 28 mycotoxins in urine.

For the urine matrix, we evaluated four different extraction solvents, including ACN, formic acid (FA) /ACN (2/98, *v*/*v*), FA/ACN (5/95, *v*/*v*), and phosphate-buffered saline (PBS) (pH = 3). As illustrated in Figure 3, when the concentration of FA increased to 2.0%, the extraction efficiencies of all toxins were above 85.0%, and AFM_1_ showed the highest extraction efficiency at 100%. When the concentration of FA increased to 5.0%, the extraction efficiencies of TEN and AME decreased from 97.0% and 95.2% to 80.0 and 79.0%, respectively. When PBS (pH = 3) was used as the extraction solvent, only AFM_1_, FBs, 3-Ac-DON, 15-Ac-DON, and TeA showed satisfactory extraction recoveries, all above 85.0%. Therefore, acetonitrile containing 2.0% formic acid was selected as the extraction solvent in this study.

### 2.2. Optimization of Sample Cleanup

**Figure 4:**
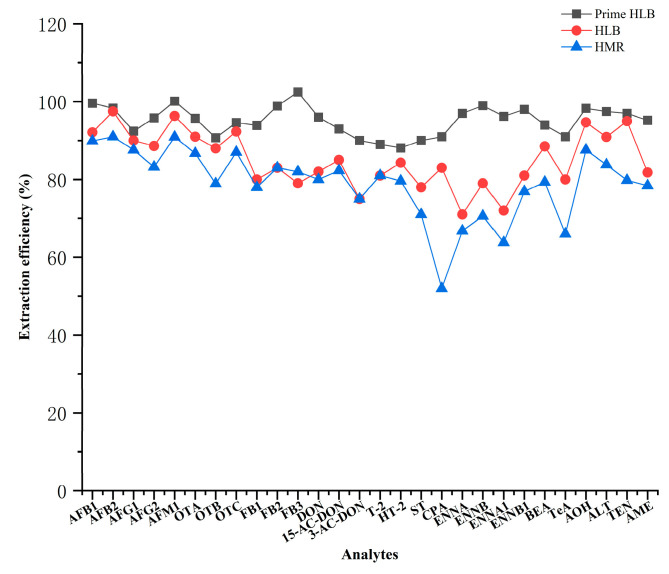
Extraction efficiencies of different SPE cartridges for 28 mycotoxins in wheat products.

**Figure 5:**
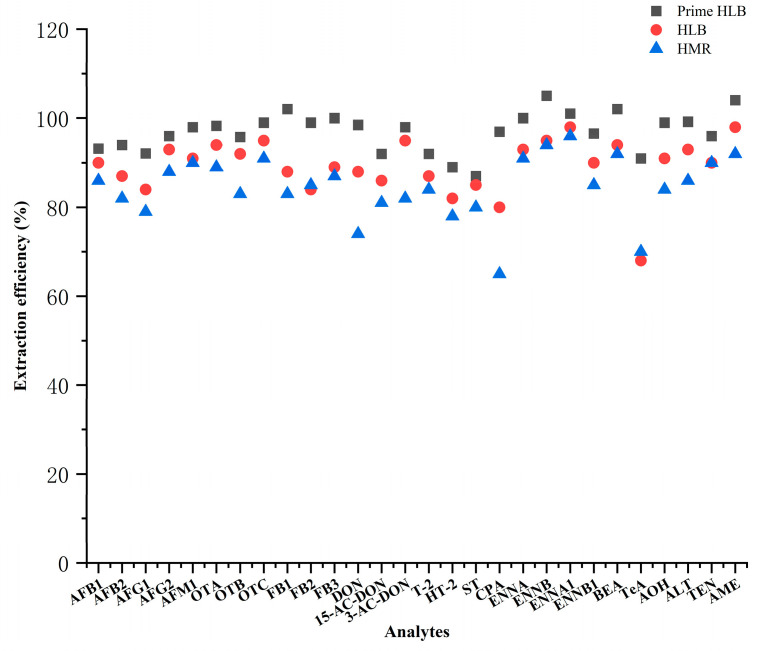
Extraction efficiencies of different SPE cartridges for 28 mycotoxins in urine.

Wheat products mainly contain proteins, lipids, pigments, and other interfering substances, whereas urine mainly contains salt, urea, and endogenous metabolites. These substances can easily cause matrix effects during analysis and, thus, affect quantitative accuracy. The SPE technique was employed to remove matrix interferences and improve method stability and accuracy. In this study, three different solid-phase extraction cartridges (Prime HLB, HLB, and HMR) were compared for their effects on the extraction recoveries of multiple mycotoxins in wheat products and urine. In both matrices, the extraction efficiencies obtained with the Prime HLB cartridge were higher than those obtained with the other two cartridges, as illustrated in Figure 4 and Figure 5. The extraction efficiencies obtained with the Prime HLB cartridge were 88.1~102% and 91.0~105%, respectively. Therefore, the Prime HLB cartridge was selected as the solid-phase extraction cartridge for sample cleanup in both matrices.

### 2.3. Optimization of Enzyme Hydrolysis

After renal metabolism, mycotoxins in urine may exist not only in free forms but also as glucuronide conjugates formed through phase II metabolism. These conjugated metabolites are more polar and cannot be fully detected by conventional methods designed for free toxins. Therefore, β-glucuronidase was added to urine samples for enzymatic hydrolysis to convert the conjugated metabolites into their free forms [23].

In this study, a 1.0 mL urine sample was treated with different volumes of β-glucuronidase, namely 10.0 µL (1000 U), 20.0 µL (2000 U), 30.0 µL (3000 U), and 40.0 µL (4000 U). After incubation in a 37 °C water bath for 16 h, the extraction efficiencies of DON and TeA increased with increasing amounts of β-glucuronidase, whereas no obvious changes were observed for the other mycotoxins. When the enzyme volume increased from 20.0 µL to 30.0 µL, no further obvious improvement in extraction efficiency was observed. These results indicated that the optimal extraction efficiency could be achieved by adding 20.0 µL β-glucuronidase per milliliter of urine sample. DON and TeA, which may occur in conjugated forms in urine, became free forms when urine samples were hydrolyzed [23]. Therefore, 20.0 µL β-glucuronidase was added to each 1.0 mL urine sample in this study.

### 2.4. Validation Experiments

The established method (enzymatic hydrolysis, SPE cleanup, and UPLC-MS/MS analysis) was evaluated in terms of linearity, recovery (RE), repeatability (RSD), limit of detection (LOD), limit of quantification (LOQ), and matrix effects (MEs). Method validation was conducted with reference to the general principles of ICH Q2(R2) for analytical procedure validation [40]. For the wheat product matrix, the analytical performance was further evaluated in light of the Commission Implementing Regulation (EU) 2023/2782 [41].

The standard stock solutions were diluted with H_2_O/ACN (1/9, *v*/*v*) to prepare mixed standard solutions. For wheat product samples, the concentration range of 0.01~400 μg/L was used. For urine samples, the concentration range was 0.001~200 μg/L. Satisfactory linearity was obtained with correlation coefficients (R^2^) greater than 0.99 for all analytes in both matrices.

For both methods, recoveries were calculated based on the internal standard method. In wheat product samples, the mean spiked recoveries ranged from 70.2% to 120%, and the RSDs ranged from 1.6% to 9.1%. In urine samples, the mean spiked recoveries ranged from 79.3% to 120%, and the RSDs ranged from 0.7% to 9.4%. The detailed RE (%) and RSD (%) values at each concentration level are presented in Table 1 and Table 2. For wheat product samples, the low spiking levels for all compounds were set as follows: AFB_1_ and AFG_1_, 0.08 μg/L; AFB_2_ and AFG_2_, 0.02 μg/L; AFM_1_, 0.1 μg/L; OTA, OTB, and OTC, 0.2 μg/L; FB_1_, FB_2_, FB_3_, and CPA, 5.0 μg/L; T-2, HT-2, DON, 3-Ac-DON, and 15-Ac-DON, 10.0 μg/L; ST, 1.0 μg/L; ENNA, ENNA_1_, and BEA, 1.0 μg/L; ENNB and ENNB_1_, 0.2 μg/L; ALT, 2.5 μg/L; AOH, 2.0 μg/L; TeA, 5.0 μg/L; TEN, 1.0 μg/L; and AME, 0.2 μg/L. For urine samples, the low spiking levels for all compounds were set as follows: AFB_1_ and AFG_1_, 0.04 μg/L; AFB_2_ and AFG_2_, 0.01 μg/L; AFM_1_, 0.05 μg/L; OTA, OTB, and OTC, 0.1 μg/L; FB_1_, FB_2_, FB_3_, and CPA, 2.5 μg/L; T-2, HT-2, DON, 3-Ac-DON, and 15-Ac-DON, 5.0 μg/L; ST, 0.5 μg/L; ENNA, ENNA_1_, and BEA, 0.05 μg/L; ENNB and ENNB_1_, 0.01 μg/L; ALT, 1.25 μg/L; AOH, 1.0 μg/L; TeA, 2.5 μg/L; TEN, 0.5 μg/L; and AME, 0.1 μg/L. For both matrices, the medium and high spiking levels for AFG1, AFB1, AFG2, AFB2, AFM1, OTA, OTB, OTC, FB1, FB2, FB3, T-2, HT-2, DON, 3-Ac-DON, 15-Ac-DON, ST, and CPA were set at 10-fold and 40-fold of the low spiking level, respectively. For ENNA, ENNA_1_, BEA, ENNB, ENNB_1_, ALT, AOH, TeA, TEN, and AME, the medium and high spiking levels were set at 2.5-fold and 10-fold of the low spiking levels, respectively, in wheat products. For ENNA, ENNA_1_, BEA, ENNB, and ENNB_1_, the medium and high spiking levels were set at 40-fold and 100-fold of the low spiking levels, respectively; for ALT, AOH, TeA, TEN, and AME, the medium and high spiking levels were set at 4-fold and 10-fold of the low spiking levels, respectively, in urine.

The LODs and LOQs were calculated by the low spiking level analyte concentrations corresponding to signal-to-noise ratios (S/N) of 3 and 10, respectively. In wheat product samples, the LODs ranged from 0.001 to 8.3 μg/kg, and the LOQs ranged from 0.002 to 25.0 μg/kg. In urine samples, the LODs ranged from 0.0001 to 1.0 μg/L, and the LOQs ranged from 0.0002 to 3.0 μg/L (Table 1 and Table 2). For the wheat product matrix, the LODs and LOQs of the method established in this study were superior to those of previously published methods. For instance, Kim et al. developed an LC–MS/MS method for 35 mycotoxins in wheat flour with LODs ranging from 0.01 to 7.50 μg/kg and LOQs ranging from 0.02 to 22.73 μg/kg [42]; Narváez et al. validated a method for 24 mycotoxins in breakfast cereals with LOQs of 0.20–12.5 μg/kg [43]. Compared with these studies, our method for wheat products exhibited lower minimum detection limits. Regarding the urine matrix, Peris-Camarasa et al. reported a UHPLC–MS/MS method for 12 urinary mycotoxins with LOQs ranging from 0.005 to 0.5 ng/mL [25]. The method developed in the present study demonstrated more favorable detection limits.

In this study, matrix effects in wheat product samples and urine samples were systematically evaluated. As shown in Table 1 and Table 2, the matrix effects ranged from 33.9% to 116% in wheat product samples and from 9.65% to 114% in urine samples. Among all analytes, AFB_1_, AFB_2_, and AFG_1_ exhibited pronounced signal suppression in both matrices. Interference from endogenous matrix components can give rise to matrix effects, resulting in signal enhancement or suppression of the target analytes. Although some target compounds exhibited matrix effects to a certain extent, the matrix-induced signal variations were effectively compensated after internal standard correction. The recoveries of all target mycotoxins ranged from 70.2% to 120%, with RSDs ranging from 0.7% to 9.4%, indicating that internal standard correction ensured the accuracy of the method.

Overall, compared with previously reported methods, the present method offers several practical advantages. It can be applied to both wheat products and human urine, covers a broad panel of 28 mycotoxins, achieves satisfactory sensitivity, and simplifies the sample preparation procedure. These features make the presented method suitable for integrating mycotoxin occurrence investigation and human biomonitoring studies.

### 2.5. Application of the Method

**Figure 6:**
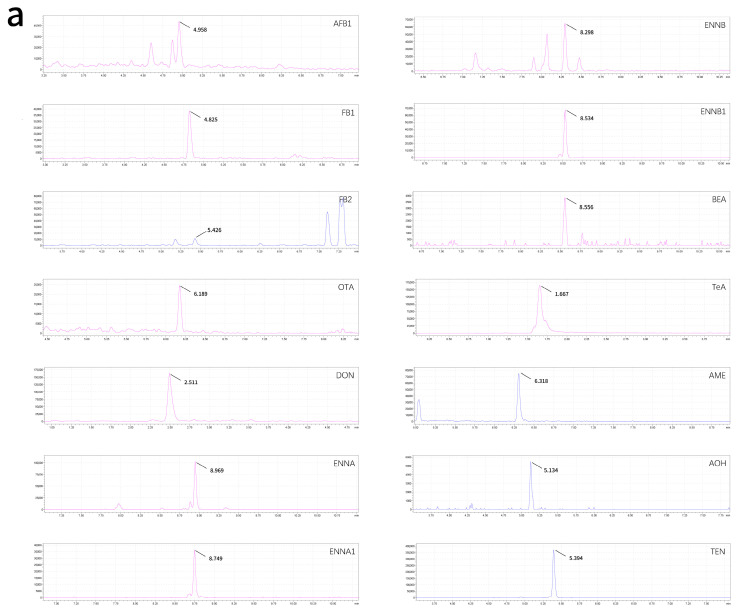

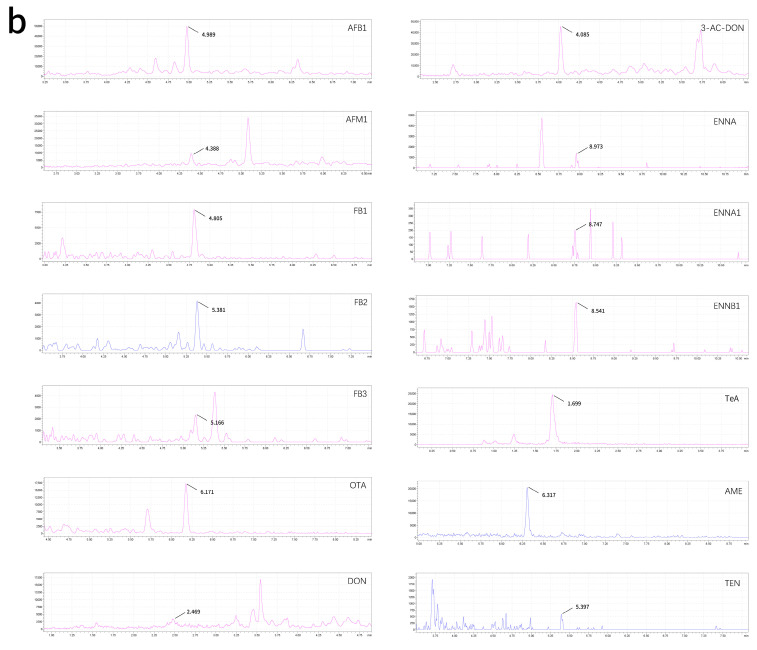
Representative MRM chromatograms of mycotoxins detected in wheat product samples (**a**) and urine samples (**b**).

The optimized method was applied to the analysis of 10 wheat product samples and 10 urine samples to evaluate its practical applicability. Quality control (QC) samples were included in all analytical batches, and the measured values were required to be within ±15% of the theoretical values. Multiple mycotoxins were detected in both matrices. In wheat product samples, at least seven mycotoxins were detected in each sample, with DON, ENNB, ENNA_1_, ENNB_1_, TeA, and TEN detected in all samples, and the concentrations of these mycotoxins ranged from 0.061 to 108.06 μg/kg. In urine samples, at least five mycotoxins were detected in each sample, and FB_1_ was detected in all samples. TeA showed the highest mean detected concentration in both matrices. The mean concentrations of wheat product and urine samples were 39.89 ± 37.18 μg/kg and 14.7 ± 14.8 μg/L, respectively. The detailed occurrence results are summarized in Table 3, and representative multiple reaction monitoring (MRM) chromatograms are shown in Figure 6. It should be noted that these real sample data were obtained from a limited number of samples and are presented only for methodological demonstration, rather than as representative contamination or population exposure levels. Nevertheless, the successful detection of multiple target analytes in both matrices confirms the practical applicability of the developed UPLC–MS/MS method to complex food and biological samples.

## 3. Conclusions

In this study, an accurate, sensitive, and efficient UPLC-MS/MS method was established for the determination of 28 mycotoxins in wheat products and human urine. The mean spiked recoveries of the target toxins ranged from 70.2% to 120% in wheat products and from 79.3% to 120% in human urine, with RSD ranges of 1.6~9.1% and 0.7~9.4%, respectively. These values were all within acceptable ranges. After optimization, the method showed good sensitivity, with LODs of 0.001~8.3 μg/kg and LOQs of 0.002~25.0 μg/kg for wheat product samples, and LODs of 0.0001~1.0 μg/L and LOQs of 0.0002~3.0 μg/L for urine samples.

The optimized method was successfully applied to the analysis of wheat products and urine, in which at least seven and five mycotoxins were detected in wheat product samples and urine samples, respectively. In this preliminary application, TeA showed the highest detected concentration in both matrices, while FB_1_ exhibited relatively high detection rates in both matrices. These findings support the practical applicability of the proposed method in real samples. Overall, the established method can be used for the simultaneous quantitative determination of 28 mycotoxins in wheat products and human urine and provides technical support for the assessment of contamination levels in wheat products and for human biomonitoring.

## 4. Materials and Methods

### 4.1. Reagents and Chemicals

Methanol, acetonitrile, formic acid, and ammonium acetate were all LC-MS-grade and were purchased from Fisher Scientific (Waltham, MA, USA). β-Glucuronidase (from abalone, 100,000 U) was purchased from ANPEL Laboratory Technologies (Shanghai, China). Anhydrous sodium dihydrogen phosphate (NaH_2_PO_4_) and anhydrous disodium hydrogen phosphate (Na_2_HPO_4_) were both HPLC-grade and were purchased from Dikma Technologies (Beijing, China). The Prime HLB (3 cc, 60 mg) and HLB (3 cc, 60 mg) solid-phase extraction cartridges, as well as the ACQUITY UPLC BEH C18 (2.1 × 100 mm, 1.7 µm) column, were purchased from Waters (Milford, MA, USA). The HMR (20 mg, 30 μm, 1 mL) solid-phase extraction cartridge was purchased from Anavo (Beijing, China). Ultrapure water was prepared using a water purification system.

An ultra-high performance liquid chromatography–tandem triple quadrupole mass spectrometer (LC-MS 30A-8060NX, Shimadzu, Kyoto, Japan), high-speed refrigerated centrifuge (5810R, Eppendorf, Hamburg, Germany), electronic balance (ME204, METTLER TOLEDO, Greifensee, Switzerland), vortex mixer (MS 3 Basic, IKA, Staufen, Germany), nitrogen evaporator (N-EVAP-24, Organomation, Berlin, MA, USA), ultrapure water system (Millipore, Burlington, MA, USA), constant-temperature shaking water bath (SW22, Julabo, Seelbach, Germany), and shaker (HS 501 D, IKA, Staufen, Germany) were used.

The standard solutions of 28 mycotoxins and 18 isotopically labeled mycotoxins standard solutions were both purchased from Qingdao Pribolab Pte. Ltd. (Qingdao, China). (AFB_2_, AFG_2_, AFB_1_, and AFG_1_, 1.0 μg/mL; OTA, OTB, and OTC, 10.0 μg/mL; ENNA, ENNA_1_, BEA, ENNB, ENNB_1_, AME, TEN, AOH, ST, TeA, ALT, AFM_1_, FB_1_, FB_2_, FB_3_, DON, 3-Ac-DON, 15-Ac-DON, CPA, T-2, and HT-2, 100 μg/mL; ^13^C_15_-AME, 40 μg/L; ^13^C_17_-AFG_1_, ^13^C_17_-AFG_2_, ^13^C_17_-AFB_1_, ^13^C_17_-AFB_2_, and ^13^C_17_-AFM_1_, 100 μg/L; ^13^C_34_-FB_1_, ^13^C_34_-FB_2_, ^13^C_33_-ENNB, ^13^C_34_-ENNB_1_, ^13^C_20_-OTA, and ^13^C_22_-TEN, 200 μg/L; ^13^C_14_-AOH, 400 μg/L; ^13^C_15_-DON, ^13^C_17_-3-Ac-DON, ^13^C_10_-TeA, ^13^C_15_-ALT, and ^13^C_45_-BEA, 1000 μg/L). All standard solutions were stored at −20 °C.

### 4.2. Samples

In this study, random urine samples were collected from 10 participants at the Physicochemical Laboratory of Beijing Chaoyang District Center for Disease Control and Prevention (Chaoyang District Health Supervision Institute), Beijing, including 3 males and 7 females. In addition, 10 wheat product samples, all of which were wheat flour from different brands, were collected from the retail markets in Chaoyang District, Beijing. All samples were stored at −20 °C. All urine samples were anonymized prior to analysis. These samples were used solely for methodological demonstration and preliminary applicability evaluation of the proposed method.

### 4.3. Preparation of Standard Solutions and Quality Control Samples

Before use, the standard solutions of the 28 mycotoxins were diluted with H_2_O/ACN (1/9, *v*/*v*) to prepare working solutions at concentrations ranging from 0.001 to 400 μg/L.

Corresponding isotopically labeled internal standards were not commercially available for some target compounds; surrogate internal standards were selected for correction based on the similarity in retention times and response behaviors. ^13^C_34_-ENNB_1_ was used as the internal standard for ENNA and ENNA_1_; ^13^C_34_-FB_2_ was used as the internal standard for FB_3_; ^13^C_20_-OTA was used as the internal standard for OTB, OTC, ST, CPA, T-2, and HT-2; and ^13^C_17_-3-Ac-DON was used as the internal standard for 15-Ac-DON. The remaining 18 mycotoxins were quantified using their corresponding internal standards.

All standard solutions at different concentrations contained 0.4 μg/L ^13^C_15_-AME; 1.0 μg/L ^13^C_17_-AFG_1_, ^13^C_17_-AFG_2_, ^13^C_17_-AFB_1_, ^13^C_17_-AFB_2_, and ^13^C_17_-AFM_1_; 2.0 μg/L ^13^C_34_-FB_1_, ^13^C_34_-FB_2_, ^13^C_33_-ENNB, ^13^C_34_-ENNB_1_, ^13^C_20_-OTA, and ^13^C_22_-TEN; 4.0 μg/L ^13^C_14_-AOH; and 10.0 μg/L ^13^C_45_-BEA, ^13^C_15_-DON, ^13^C_17_-3-Ac-DON, ^13^C_10_-TeA, and _13_C^15^-ALT.

The QC samples were prepared by spiking the above mixed standard solution into analyte-free blank samples to obtain three concentration levels (low, medium, and high). QC samples were analyzed in each batch of test samples, and the measured values were required to be within ±15% of the theoretical values.

To monitor potential background contamination during sample preparation and instrumental analysis, solvent blanks and procedural blanks were included in each analytical batch. The procedural blanks consisted of blank matrix samples and were subjected to the same extraction, cleanup, and UPLC–MS/MS analytical procedures as the test samples, but without the addition of target analytes. The solvent blanks were used to evaluate potential contamination and interference originating from the solvents and the instrumental system. No obvious interfering peaks corresponding to the target analytes were observed in the blank samples.

### 4.4. Sample Preparation

Wheat product samples: Accurately weigh 2.00 g of the ground sample (to 0.01 g) into a 50.0 mL centrifuge tube, add the internal standards, and vortex-mix thoroughly. Then, add 10.0 mL of 1.0% formic acid H_2_O/ACN (10/90, *v*/*v*) to the mixed extraction solvent. After vortex mixing, extract the sample by shaking for 30 min. Following centrifugation at 10,000 r/min for 10 min at 4 °C, 1.0 mL of the supernatant is collected for cleanup.

Urine samples: The urine samples were thawed at room temperature and centrifuged at 10,000 r/min for 10 min at 4 °C. Then, 1.0 mL of urine was transferred into a 5.0 mL centrifuge tube, and the internal standards were added, followed by vortex mixing. After the addition of 20.0 µL β-glucuronidase (from abalone), the mixture was vortex-mixed and enzymatically hydrolyzed in a 37 °C water bath for 16.0 h. After the mixture was cooled to room temperature, 1.0 mL of FA/ACN (2/98, *v*/*v*) extraction solvent was added, followed by vortex mixing, and the mixture was allowed to stand for 30 min. After centrifugation at 10,000 r/min for 10 min at 4 °C, the supernatant was collected for cleanup.

The supernatants of both wheat product and urine samples were subjected to cleanup using a Prime HLB solid-phase extraction cartridge, separately. After sample loading, the cartridge pass-through was collected. The cartridge was then eluted sequentially with 1.0 mL of acetonitrile and 1.0 mL of methanol. The pass-through fraction and the cartridge eluates were combined and evaporated to near dryness under a stream of nitrogen in a 37 °C water bath. The residues were reconstituted with ACN/H_2_O (1/9, *v*/*v*) to final volumes of 200 μL for wheat product samples and 500 μL for urine samples. After vortex mixing for 1 min and centrifugation at 10,000 r/min for 10 min, the supernatants were collected for analysis.

For the determination of free target toxins, β-glucuronidase (from abalone) was not added to the sample; the enzymatic hydrolysis step was omitted, and all other procedures remained the same.

### 4.5. LC-MS/MS Condition

Analyses were performed using the UPLC-MS/MS system, consisting of an LC-30A (Shimadzu, Kyoto, Japan) binary pump and automatic injector coupled to an LCMS-8060NX triple quadruple mass spectrometer (Shimadzu, Kyoto, Japan).

#### 4.5.1. Chromatographic Condition

Separation was achieved on a 2.1 × 100 mm, 1.7 µm BEH C18 column (Waters, Milford, MA, USA). The flow rate was 0.3 mL/min, the column temperature was 40 °C, and the injection volume was 10.0 μL. For the target compounds (AFB_1_, AFB_2_, AFG_1_, AFG_2_, AFM_1_, OTA, OTB, OTC, FB_1_, FB_2_, FB_3_, HT-2, T-2, DON, 3-Ac-DON, 15-Ac-DON, ST, and CPA), mobile phase 1 consisted of A (0.1% formic acid in water) and B (acetonitrile). For the target compounds (ENNA, ENNA_1_, ENNB, ENNB_1_, BEA, TeA, AOH, ALT, TEN, and AME), mobile phase 2 consisted of A (5.0 mmol/L ammonium acetate aqueous solution) and B (acetonitrile). The gradient program was as follows: 0–1.00 min, 10% B; 1.00–8.00 min, 10–95% B; 8.00–12.00 min, 95% B; 12.00–12.10 min, 95–10% B; and 12.10–16.00 min, 10% B.

#### 4.5.2. Mass Spectrometry Condition

**Figure 7:**
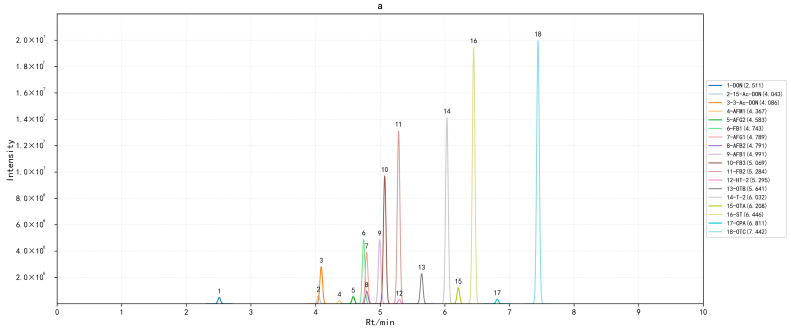

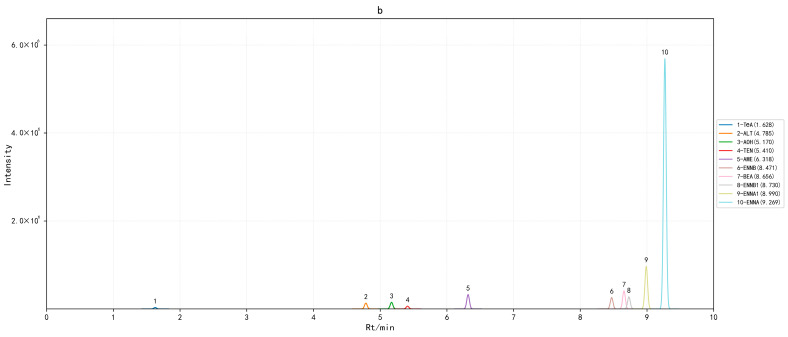
MRM chromatograms of (**a**) a standard mixture of 18 mycotoxins (100 μg/L FB_1_, FB_2_, FB_3_, CPA; 4.0 μg/L OTA, OTB, OTC; 200 μg/L DON, 3-Ac-DON, 15-Ac-DON, T-2, HT-2; 1.6 μg/L AFB_1_, AFG_1_; 0.4 μg/L AFB_2_, AFG_2_; 2.0 μg/L AFM_1_; 20.0 μg/L ST) and (**b**) a standard mixture of 10 mycotoxins (1.0 μg/L ENNB, ENNB_1_, AME; 5.0 μg/L ENNA, ENNA_1_, BEA, TEN; 12.5 μg/L ALT; 25.0 μg/L TeA; 10.0 μg/L AOH).

Both positive and negative ionization modes were run simultaneously, and MRM was employed during infusion. Other ionization source parameters were set as follows: interface temperature (I) was 300 °C, desolvation temperature was 526 °C, heat block temperature (H) was 400 °C, nebulizing gas flow (N) was 3.0 L/min, drying gas flow (G) was 5.0 L/min, heating gas flow (F) was 10.0 L/min, and DL temperature (D) was 250 °C. Other MS/MS acquisition conditions are provided in Table 4. Figure 7 shows the chromatograms of the 28 target compounds in the standard solution.

### 4.6. Method Validation

The performance of the developed method, comprising enzymatic hydrolysis, SPE cleanup, and UPLC-MS/MS analysis, was assessed by evaluating linearity, recovery (RE), repeatability (RSD), limit of detection (LOD), limit of quantification (LOQ), and matrix effects (MEs). Method validation was performed according to the general principles outlined in ICH Q2(R2) for analytical procedure validation [40]. In addition, the analytical performance for the wheat product matrix was considered in light of the Commission Implementing Regulation (EU) 2023/2782 [41].

The linearity of the different compounds was evaluated over the range of 0.001–400 μg/L. The LOD and LOQ were assessed from the results obtained for the analytes at the low spiking level, corresponding to signal-to-noise ratios (S/N) of 3 and 10, respectively. Using the internal standard correction method, the RE and RSD at low, medium, and high concentration levels were determined using six replicate blank samples. MEs were evaluated by comparing the peak areas of the analytes in blank matrix-matched standard solutions with those in pure solvent standard solutions. RE was evaluated by comparing the peak areas of the analytes in blank matrix samples spiked before sample pretreatment with those in blank matrix samples spiked after sample pretreatment. Based on the above validation principles, the obtained performance characteristics were considered acceptable for the intended application of this method in wheat products and urine samples.

## Figures and Tables

**Table 1:** Linear range, recovery, repeatability, LOD, LOQ and ME of 28 kinds of mycotoxins in wheat products (*n* = 6).

Analyte	Linear Range (μg/L)	RE (%)			RSD (%)	LOQ (μg/kg)	LOD (μg/kg)	ME (%)
		Low	Medium	High				
AFB_1_	0.04~3.20	79.8	78.0	96.4	2.2~3.9	0.02	0.01	47.7
AFB_2_	0.01~0.80	85.6	105	114	4.7~7.4	0.1	0.03	33.9
AFG_1_	0.04~3.20	86.0	102	119	6.3~7.4	0.4	0.1	43.9
AFG_2_	0.01~0.80	91.1	105	114	4.4~5.9	0.1	0.03	68.7
AFM_1_	0.05~4.00	92.1	93.6	106	3.4~6.8	0.25	0.08	82.9
OTA	0.1~8.00	112	120	111	2.4~8.9	0.5	0.2	100
OTB	0.1~8.00	76.0	105	109	2.8~6.7	0.15	0.05	107
OTC	0.1~8.00	76.4	104	95.8	3.2~7.1	0.05	0.02	98
FB_1_	2.5~200	115	116	110	4.8~6.6	0.25	0.08	110
FB_2_	2.5~200	86.9	92.8	100	4.1~6.2	0.25	0.08	106
FB_3_	2.5~200	70.2	80.8	99.5	3.1~5.3	0.25	0.08	116
T-2	5.0~400	76.3	87.9	88.5	3.7~7.6	1.25	0.4	116
HT-2	5.0~400	104	111	111	2.1~6.0	20.0	6.7	93
DON	5.0~400	119	109	110	4.9~8.2	1.25	0.4	85.4
3-Ac-DON	5.0~400	89.8	91.4	94.8	5.1~8.8	5.0	1.7	87.0
15-Ac-DON	5.0~400	96.1	102	111	4.2~7.6	25.0	8.3	82.4
ST	0.5~40.0	97.2	104	108	2.5~4.6	0.16	0.05	108
CPA	2.5~200	72.2	70.2	72.4	1.6~4.6	20.0	6.7	95.3
ENNA	0.05~10.0	115	113	103	4.5~7.7	0.025	0.008	115
ENNA_1_	0.05~10.0	111	113	118	2.7~9.1	0.025	0.008	108
ENNB	0.01~2.0	113	108	115	3.7~7.8	0.002	0.001	114
ENNB_1_	0.01~2.0	109	117	118	3.5~7.4	0.002	0.001	116
BEA	0.05~10.0	82.5	96.7	120	4.6~6.0	0.05	0.02	109
ALT	1.25~25.0	87.2	105	102	2.0~4.8	1.25	0.4	110
AOH	1.0~20.0	93.5	108	105	5.9~7.3	0.3	0.1	86.1
TeA	2.5~50.0	93.4	103	103	3.2~4.8	0.25	0.08	92.7
TEN	0.5~10.0	86.4	104	95.7	2.4~5.0	0.02	0.01	97
AME	0.1~2.0	89.6	104	114	1.9~2.2	0.03	0.01	99

**Table 2:** Linear range, recovery, repeatability, LOD, LOQ and ME of 28 kinds of mycotoxins in urine (*n* = 6).

Analyte	Linear Range (μg/L)	RE (%)			RSD (%)	LOQ (μg/L)	LOD (μg/L)	ME (%)
		Low	Medium	High				
AFB_1_	0.004~1.60	83.2	105	106	2.1~4.1	0.001	0.0003	10.9
AFB_2_	0.001~0.40	116	104	100	3.2~6.4	0.0025	0.0008	23.3
AFG_1_	0.004~1.60	100	96.1	81.7	1.8~2.5	0.001	0.0003	9.65
AFG_2_	0.001~0.40	92.1	119	100	4.6~9.4	0.0025	0.0008	82.7
AFM_1_	0.005~2.00	118	95.1	94.5	3.8~7.9	0.00125	0.0004	89.9
OTA	0.01~4.00	83.6	103	98.3	1.6~3.7	0.003	0.001	87.3
OTB	0.01~4.00	79.7	89.8	79.3	3.4~5.7	0.004	0.001	83.0
OTC	0.01~4.00	89.4	99.0	99.3	2.6~5.1	0.004	0.001	94.4
FB_1_	0.25~100	116	102	105	1.6~5.7	0.05	0.02	91.7
FB_2_	0.25~100	113	98.0	99.0	1.9~3.7	0.025	0.008	103
FB_3_	0.25~100	120	96.5	101	1.4~7.1	0.03	0.01	93.9
T-2	0.50~200	94.3	92.4	95.6	1.5~3.1	0.2	0.07	83.7
HT-2	0.50~200	94.0	97.2	94.3	2.6~8.4	0.6	0.2	87.4
DON	0.50~200	98.4	109	108	4.3~5.6	1.2	0.4	97.6
3-Ac-DON	0.50~200	105	103	101	3.9~5.0	1.0	0.3	94.2
15-Ac-DON	0.50~200	107	106	108	2.9~7.0	0.6	0.2	85.9
ST	0.05~20.0	86.2	95.0	96.0	1.0~2.9	0.05	0.02	97.1
CPA	0.25~100	103	98.0	101	1.0~2.0	3.0	1.0	113
ENNA	0.005~5.0	99.2	100	101	2.8~4.1	0.0008	0.0002	99.0
ENNA_1_	0.005~5.0	98.9	103	106	2.6~3.1	0.0005	0.0001	84.2
ENNB	0.001~1.0	100	103	105	0.7~4.6	0.0002	0.0001	99.8
ENNB_1_	0.001~1.0	99.2	97.1	101	1.6~4.2	0.0002	0.0001	83.8
BEA	0.005~5.0	96.6	93.1	101	1.2~3.1	0.0006	0.0002	90.1
ALT	0.125~12.5	99.3	111	99.2	2.3~8.9	0.07	0.02	94.4
AOH	0.10~10.0	107	109	104	3.0~6.4	0.04	0.01	96.6
TeA	0.25~25.0	115	107	101	3.6~8.2	0.5	0.2	114
TEN	0.05~5.0	100	92.0	103	3.4~8.6	0.01	0.003	109
AME	0.01~1.0	106	108	104	2.2~4.6	0.003	0.001	93.0

**Table 3:** Occurrence of mycotoxins in wheat products and urine samples with enzyme treatment (*n* = 10).

Analytes	Wheat Products				Urine			
	Positive (%)	Mean ± SD (μg/kg)	Range (μg/kg)	Median (μg/kg)	Positive (%)	Mean ± SD (μg/L)	Range (μg/L)	Median (μg/L)
AFB_1_	30%	0.008 ± 0.003	0.005~0.01	0.01	50%	0.0119 ± 0.0022	0.0086~0.0139	0.0128
AFM_1_	ND				50%	0.0742 ± 0.0447	0.0383~0.1494	0.0564
FB_1_	80%	0.302 ± 0.288	<LOD~0.96	0.225	100%	0.10 ± 0.02	0.07~0.12	0.10
FB_2_	30%	0.10 ± 0.06	<LOD~0.15	0.12	90%	0.019 ± 0.009	0.010~0.033	0.016
FB_3_	ND				20%	0.017 ± 0.0014	0.016~0.018	0.017
OTA	30%	0.43 ± 0.31	0.25~0.79	0.25	10%	0.022	0.022	0.022
DON	100%	31.59 ± 23.53	4.90~85.60	25.95	60%	2.2 ± 1.7	0.6~5.5	1.9
3-Ac-DON	ND				40%	2.5 ± 1.5	1.0~4.6	2.3
ENNA_1_	100%	0.510 ± 0.582	0.061~1.629	0.211	20%	0.001	0.001	0.001
ENNA	80%	0.126 ± 0.124	0.013~0.372	0.074	40%	0.00035 ± 0.00013	0.0002~0.0005	0.00035
ENNB_1_	100%	0.978 ± 1.069	0.064~2.627	0.372	60%	0.0022 ± 0.0003	0.0020~0.0028	0.0022
ENNB	100%	2.365 ± 2.960	0.079~7.748	1.018	ND			
BEA	20%	0.31 ± 0.12	0.22~0.39	0.31	ND			
TeA	100%	39.89 ± 37.18	1.09~108.06	18.68	90%	14.7 ± 14.8	0.3~43.4	11.9
AOH	70%	0.7 ± 1.0	0.15~2.7	0.15	ND			
TEN	100%	5.83 ± 6.72	0.21~18.52	2.93	60%	0.024 ± 0.013	0.012~0.045	0.023
AME	90%	0.60 ± 0.77	0.13~2.53	0.29	70%	0.019 ± 0.018	0.003~0.044	0.010

For calculation of mean, standard deviation and median values, a concentration < LOD was assigned half the LOD.

**Table 4:** MRM transitions and MS/MS parameters for 28 target compounds.

Analyte	Time (min)	Precursor Ion (*m*/*z*)	Product Ion (*m*/*z*)	Q1 (V)	CE (V)	Q3 (V)
TeA	1.628	196.1	112 * 139	11 11	23 19	19 27
DON	2.511	297	249.1 * 203.1	−14 −11	−9 −16	−17 −24
15-Ac-DON	4.043	339	137.1 * 321	−16 −17	−19 −12	−30 −24
3-Ac-DON	4.086	339	231.1 * 203	−17 −15	−16 −19	−27 −21
AFM_1_	4.367	329	273 * 229	−16 −16	−24 −40	−19 −24
AFG_2_	4.583	331	245 * 257	−16 −16	−30 −32	−17 −19
FB_1_	4.743	722.2	352.2 * 334.4	−20 −20	−36 −40	−25 −13
ALT	4.785	291.1	186.1 * 214.1	17 11	26 25	20 19
AFG_1_	4.789	329	243 * 283	−16 −16	−27 −25	−17 −20
AFB_2_	4.791	315	259 * 287	−15 −15	−30 −25	−13 −20
AFB_1_	4.991	313	241 * 285	−15 −15	−35 −23	−17 −20
FB_3_	5.069	706.2	336.3 * 318.2	−20 −20	−38 −40	−16 −22
AOH	5.170	257	147.1 * 159.1	14 14	33 36	29 15
FB_2_	5.284	706.3	336.4 * 318.25	−20 −20	−36 −40	−24 −16
HT-2	5.295	425	263.1 * 245	−16 −12	−12 −15	−18 −16
TEN	5.410	413.1	214.1 * 271.2	23 13	25 19	24 28
OTB	5.641	370.1	205 * 187	−26 −26	−22 −36	−22 −21
T-2	6.032	484.3	185.1 * 305	−11 −11	−19 −14	−13 −22
OTA	6.208	404.1	239 * 358	−11 −11	−22 −16	−26 −26
AME	6.318	271.1	256.1 * 228.1	15 15	23 28	25 14
ST	6.446	325.1	310.1 * 281.1	−12 −16	−24 −35	−23 −20
CPA	6.811	335.05	139.7 * 179.75	19 19	27 27	24 30
OTC	7.442	432	358 * 239	−16 −22	−17 −26	−14 −18
ENNB	8.471	640.4	86.1 * 196.2	−32 −32	−55 −26	−17 −14
ENNB_1_	8.730	654.5	196.2 * 210.2	−24 −24	−24 −23	−14 −15
BEA	8.656	784.4	244.1 * 134.1	−28 −40	−32 −55	−28 −24
ENNA_1_	8.990	668.5	210.15 * 100.1	−24 −24	−23 −54	−15 −11
ENNA	9.269	699.1	210.2 * 228.3	−20 −24	−33 −36	−14 −27
^13^C_10_-TEA	1.629	206.1	144.9	15	16	13
^13^C_15_-DON	2.487	312	263.1	−15	−14	−26
^13^C_17_-3-Ac-DON	4.086	356	245	−17	−17	−17
^13^C_17_-AFM_1_	4.366	346.1	288.1	−17	−24	−21
^13^C_17_-AFG_2_	4.580	347.9	329.9	−17	−26	−24
^13^C_34_-FB_1_	4.743	756.5	356.4	−22	−42	−26
^13^C_15_-ALT	4.784	306	226.8	17	25	10
^13^C_17_-AFG_1_	4.786	346	257	−17	−26	−18
^13^C_17_-AFB_2_	4.788	332	272.9	−16	−30	−20
^13^C_17_-AFB_1_	4.988	329.9	254.9	−16	−37	−18
^13^C_14_-AOH	5.172	271	226.9	14	27	24
^13^C_34_-FB_2_	5.285	740.4	358.4	−20	−38	−26
^13^C_22_-TEN	5.408	434.8	286.7	23	30	18
^13^C_20_-OTA	6.205	424.1	250	−12	−24	−18
^13^C_15_-AME	6.317	286.05	269.85	15	24	27
^13^C_33_-ENNB	8.469	673.5	207.3	−20	−27	−15
^13^C_45_-BEA	8.656	829.4	259.3 * 143.3	−24 −24	−27 −55	−10 −25
^13^C_34_-ENNB_1_	8.727	688.9	207.1 * 225.3	−20 −24	−25 −32	−22 −12

* Quantifier transition.

## Data Availability

The original contributions presented in this study are included in the article. Further inquiries can be directed to the corresponding authors.
